# Identifying Usual Food Choice Combinations With Walnuts: Analysis of a 2005–2015 Clinical Trial Cohort of Overweight and Obese Adults

**DOI:** 10.3389/fnut.2020.00149

**Published:** 2020-09-23

**Authors:** Vivienne X. Guan, Elizabeth P. Neale, Linda C. Tapsell, Yasmine C. Probst

**Affiliations:** ^1^Faculty of Science, Medicine and Health, School of Medicine, University of Wollongong, Wollongong, NSW, Australia; ^2^Illawarra Health and Medical Research Institute, University of Wollongong, Wollongong, NSW, Australia

**Keywords:** food choices, walnuts, obesity, clinical trial, data mining

## Abstract

Consumption of nuts has been associated with a range of favorable health outcomes. Evidence is now emerging to suggest that walnuts may also play an important role in supporting the consumption of a healthy dietary pattern. However, limited studies have explored how walnuts are eaten at different meal occasions. The aim of this study was to explore the food choices in relation to walnuts at meal occasions as reported by a sample of overweight and obese adult participants of weight loss clinical trials. Baseline usual food intake data were retrospectively pooled from four food-based clinical trials (*n* = 758). A nut-specific food composition database was applied to determine walnut consumption within the food intake data. The *a priori* algorithm of association rules was used to identify food choices associated with walnuts at different meal occasions using a nested hierarchical food group classification system. The proportion of participants who were consuming walnuts was 14.5% (*n* = 110). The median walnut intake was 5.14 (interquartile range, 1.10–11.45) g/d. A total of 128 food items containing walnuts were identified for walnut consumers. The proportion of participants who reported consuming unsalted raw walnut was 80.5% (*n* = 103). There were no identified patterns to food choices in relation to walnut at the breakfast, lunch, or dinner meal occasions. A total of 24 clusters of food choices related to walnuts were identified at others (meals). By applying a novel food composition database, the present study was able to map the precise combinations of foods associated with walnuts intakes at mealtimes using data mining. This study offers insights into the role of walnuts for the food choices of overweight adults and may support guidance and dietary behavior change strategies.

## Introduction

Nut consumption is recommended in food-based dietary guidelines across the globe (1). Habitual nut consumption has been associated with a reduced risk of metabolic syndrome and type 2 diabetes mellitus (2–8), with emerging evidence suggesting that nuts may play an important role in supporting the consumption of a healthy dietary pattern (9, 10). However, there is a large shortfall in optimal intake of nuts. A study examining dietary trends from 1990 to 2017 in 195 countries revealed that, on average, people ate only 12% of the recommended amount of nuts and seeds, which equated to ~3 g/d, compared with the 21 g recommended per day (11). In Australia, nut intake was estimated at an average of 4.61 g/d, with only 5.6% of people consuming the recommended daily amount of nuts (12). As a result intervention studies have been developed to determine strategies for encouraging increased nut consumption.

The effectiveness of a range of population-level dietary intervention has been systematically evaluated over the past decade. Several promising interventions, such as mass media campaigns, food labeling, and food pricing strategies, have been identified (13). However, there is no consistent evidence on the effectiveness of these interventions on nuts consumption (11). Thus, understanding the role of nuts, which make up the recommended dietary patterns, is required to identify the challenges and opportunities to improving dietary intake of nuts. Given meal patterns are more likely to reflect eating (14), actual foods that comprised the meal-based intakes offer practical opportunities for dietary advice (15). Therefore, exploring how food choices are constructed in relation to nuts at meal occasions may play an important role in translating dietary recommendations for practical food choices of nuts. However, limited research has been conducted in this area.

A challenge to exploring food choices in relation to nuts at meals appears to be the interindividual and intraindividual variation in choosing different types of foods and the frequency of food intakes. An inaccessible number of food choice combinations may be created because of variation in food choices, which will be addressed by this research. Literature has suggested that exploring single foods that form a food combination offer a more precise food consumption distributions to represent dietary intake (16–19). Taking into account the time intervals of eating (for example, daily vs. weekly) while identifying closely related food groups at meals tends to improve the accuracy of intake estimation by comparisons to disaggregation alone.

Advances in data analysis techniques may provide alternative methods to overcome the challenges for exploring food choice within meals. The *a priori* algorithm has been used to explore food combination patterns at meal occasions in many studies (18, 20–23). The outcomes of the analyses are formed to suggest closely related food items at meal occasions. For example, at the lunch meal occasion, if cheese and ham were reported as consumed, then bread was also consumed. Knowing such relationships may help to personalize nutrition counseling for clients. Suggesting strategies for what clients can do practically is one strategy to increase the intake frequency of less frequently consumed foods, such as walnuts. The identified closely related food items within food combinations may also be used to suggest foods to be eaten together to improve dietary adherence to national recommendations, such as total nut intake.

Exploring a specific type of nuts in diets may provide insights into dietary habits in relation to nuts for dietary recommendations. The most produced and most consumed tree nuts globally are almonds, cashews, hazelnuts, pistachios, and walnuts (24). Although walnuts are rich in unsaturated fats, minerals, vitamins, fiber, and polyphenols, they stand out for their high polyunsaturated fatty acids content (25). Literature has suggested that the intake of walnuts was associated with decreasing the risk of cardiometabolic diseases (26–30). Thus, the aim of this study was to explore the food choices in relation to walnuts at meal occasions as reported by a sample of overweight and obese volunteers in weight loss trials.

## Materials and Methods

### Study Participants

This study used data from four previously published food-based clinical trial studies (9, 31–33). All studies were registered with the Australian Clinical Trials Registry (ACTRN12608000453381, ACTRN12608000425392, ACTRN12610000784011, and ACTRN 12614000581662). The data from the four studies were linked and retrospectively pooled for analysis as a baseline cohort as reported here. Ethics approval was obtained for the initial studies and for this investigation. The original studies were food-based randomized controlled trials for weight loss, conducted between 2005 and 2015: study 1 (2005–2006) (31), study 2 (2009–2010) (32), study 3 (2010–2012) (33), and study 4 (2014–2015) (9) in the University of Wollongong, Australia. All study participants were recruited from the Illawarra, a major coastal region 70 km south of Sydney, Australia. The World Health Organization Body Mass Index (BMI) classifications were used to determine overweight (BMI of ≥25 kg/m^2^) and obese (BMI of ≥30 kg/m^2^) participants (34).

Detailed study protocol information and inclusion and exclusion criteria are presented in Supplementary Table 1. In brief, all trials had similar inclusion criteria, including overweight and obese female and male adults with a stable body weight prior to the trial. Exclusion criteria included inadequate conversational English for all studies, major illness (e.g., cancer) and chronic diseases (e.g., diabetes) for studies 1–3, and severe medical conditions for study 4.

### Anthropometric Variables

Anthropometric variables were measured by trained research staff using standardized protocols. Height was measured using a stadiometer without shoes for all studies. Body weight (kg) was measured in an upright position (in minimal clothing with no shoes) using digital scales with a bioelectrical impedance component (Tanita TBF-662, Wedderburn Pty Ltd., Ingleburn, NSW, Australia).

### Dietary Intake Data and Food Intake Data Preparation

Self-reported food intake data were assessed from a dietitian-administered diet history interview, which was validated previously (35). Participants were asked to recall their intakes during the interview, reflecting on usual weekly consumption recorded on the basis of participant-defined meal occasions. A food checklist was applied to capture commonly omitted food items during the interview. The food intake data were sorted to align with a 7-days equivalence to a weekly intake pattern based on the reported intake frequencies.

To evaluate potential underreporting of energy intake in this study, the Goldberg method was used (36). Self-reported energy intake (rEI) was divided with predicted basal metabolic rate (pBMR) values (36). pBMR was calculated by using age, weight, and sex as per the Mifflin equation (37). An rEI/pBMR value of 1.35 was therefore set as the cutoff value to determine potential underreporting of energy intake in this study (36, 38). Participants were categorized as plausible reporters and underreporters.

The dietary data in the four studies were analyzed using FoodWorks Professional nutrient analysis software (Xyris Software, Spring Hill, QLD, Australia). Different versions of FoodWorks were originally used (version 3 using the AUSNUT 1999 food composition database for study 1, version 6 using the AUSNUT 2007 food composition database for studies 2 and 3, and version 7 using the AUSNUT 2007 food composition database for study 4). Because the values of dietary intake measurements used are likely to influence the results, the more recent release of the AUSNUT 2011–13 food composition database was used for the analyses of this study to standardize dietary data (39). For this to occur, a previously developed matching file (40, 41) and “umbrella foods” list (41) were used to translate the food items from the AUSNUT 1999 and 2007 to the AUSNUT 2011–13 food composition database. All of the food matching was performed using the VLOOKUP function in Microsoft Excel (Microsoft Corporation, 2010, version 14.0.7). In order to determine walnut consumption, a nut-specific food composition database (42) was applied to the dietary intake data. Walnuts are commonly consumed alone or as part of mixed foods, such as carrot cake and brownies (42). The detailed food intake data preparation process has been reported elsewhere (23). In brief, major (*n* = 24), submajor (*n* = 132), and minor (*n* = 515) food groups in the AUSNUT 2011–13 food classification system (25, 39) were applied in the present study. For the purpose of food-level analyses, food intake was grouped into participant-defined meals, which were breakfast, lunch, and dinner. Other than “breakfast,” “lunch,” and “dinner,” such as morning tea, afternoon tea, desserts, extras, and snacks, were grouped into an “other” meal occasion. Food items at the submajor and minor food group levels were examined (23). To prevent undue duplication, repeated food items at each food group level within meals were removed from the dataset. It aimed to make sure that each food group was included only once for each meal occasion at each food level. RStudio, version 1.0.44 (incorporating R, version 3.2.5; The R Foundation for Statistical Computing, Vienna, Austria), was used to explore the frequencies of individual food groups within the meals (43).

### Statistical Analysis

Data extracted from the trials included the diet history data at baseline after randomization for studies 1, 2, and 3 and prior to randomization for study 4. The baseline body weight, baseline BMI, age, gender, education level, smoking status, and physical activity level data were also collated. Participants with missing data for education and physical activity were excluded from the analysis. Participants aged <19 years were also excluded. The final number of participants included in the analysis was 758. Normality of the pooled cohort data was tested using the Shapiro–Wilk test and visually inspected using a histogram and normal Q–Q plot. Baseline characteristics for the four study samples were summarized and compared using one-way analysis of variance for parametric continuous variables with *post-hoc* comparisons using Bonferroni adjustment and the Kruskal–Wallis H test for non-parametric distributed variables. The Pearson χ^2^ statistic was used to compare the proportions with *post-hoc* comparisons conducted using Bonferroni-adjusted *z* tests to compare the columns.

Data mining techniques were used to identify food groups associated with walnut consumption, using RStudio, version 1.0.44 (incorporating R, version 3.2.5; The R Foundation for Statistical Computing, Vienna, Austria) (43). The detailed analysis method is described elsewhere (23). In brief, by letting I = I1, I2, …. In a set of binary attributes, called items were developed. In this case, items are considered to be the food items. By letting *D* be a database of transactions, each transaction *d* indicates the dietary intake record reported by one participant. Each transaction d is represented as a binary vector, with *d*[*k*] = 1 if a participant consumed the food item Ik, and *d*[*k*] = 0 if a participant did not consumer the food item (44, 45). In general, a set of food items (X or Y) is a set of some items in I. A transaction *d* satisfies X if for all items Ik in X, *t*[*k*] = 1 (44, 45). An association rule is a pair (X, Y) of sets of attributes, denoted by XY (44, 45), and is presented to indicate that if the antecedent X happens (also called the left-hand-side), the consequent Y also happens (also called the right-hand side), where *X, Y*⊆*I* (44, 45).

A two-step descriptive method, the *a priori* algorithm of association rules was used in the present analysis (44, 45). First, a set of frequent item sets is generated. Second, the frequent item sets are used to generate association rules. Constraints, such as the support, confidence, and lift are applied to identify the interested association rules. Support is a frequency threshold that represents the percentage of the containing identified frequent item sets, X and Y (44, 45) (Equation 1). The confidence of a rule is the percentage of records D containing both antecedent X and consequent Y (Equation 2) (44, 45). Support and confidence are used to reflect the strength of an identified rule, where higher values imply a stronger relationship for the identified association rule (44, 45). Lift was applied to assess the dependency between antecedent and consequent (46) (Equation 3). A lift >1 indicates that antecedent and consequent food items are more likely to depend on each other.

(1) $$\begin{array}{r} support\left(X\Rightarrow Y\right)=\;P(X\cup Y) \end{array}$$

(2) $$\begin{array}{r} \text{confidence}\left(\text{X}\Rightarrow \text{Y}\right)\text{=}\frac{\text{support }(\text{X}\cup \text{Y})}{\text{support }(\text{X})} \end{array}$$

(3) $$\begin{array}{r} \text{lift }(\text{X}\Rightarrow \text{Y})\text{=}\frac{\text{support }(\text{X}\cup \text{Y})}{\text{support }(\text{X})\;\times \text{ support }(\text{Y})} \end{array}$$

In the analyses reported here, the threshold of the possible food group combinations at events (meals) was set as one-quarter of study participants in the cohort (47). In other words, at least 25% of participants would need to have reported a specific combination of food groups in relation to walnuts for the food combination to be reported in the present analysis. The default value for the *a priori* algorithm within the R software (0.80) was used for the values of confidences (43). At each event, many closely related food groups may be identified in the dataset, contributed by inherent variability related to food intakes. Thus, redundant closely related food groups were removed to minimize unnecessary complexity by comparing closely related food groups at events (46, 48).

## Results

Data for 758 participants were analyzed (Table 1). The proportion of participants who were consuming walnuts was 14.5% (*n* = 110). The median walnut intake was 5.14 (interquartile range, 1.10–11.45) g/d. A total of 128 food items containing walnuts were identified for walnut consumers. The proportion of participants who reported unsalted raw walnut was 80.5% (*n* = 103). Walnuts were also consumed as carrot cake (*n* = 14) and in a brownie with nuts (*n* = 11). Walnuts were consumed at breakfast (*n* = 18, 14.1%), lunch (*n* = 13, 10.1%), dinner (*n* = 7, 5.5%), and other meals (*n* = 90, 70.3%). The details of the food items containing walnuts at meals are shown in Table 2.

**Table 1:** Characteristics of participants by clinical trial study.

**Clinical trial (year)**	**Study 1 (2005)**	**Study 2 (2009)**	**Study 3 (2010)**	**Study 4 (2014–2015)**	***P*-value**
	***n* = 103**	***n* = 111**	***n* = 111**	***n* = 433**	
Age group, *n* (%)					0.696
20–34 years	16 (15.54)	12 (10.81)	7 (6.31)	78 (18.02)	
35–49 years	48 (46.60)	66 (59.46)	46 (41.44)	235 (54.27)	
50–64 years	38 (36.89)	33 (29.73)	55 (49.55)	120 (27.71)	
≥65 years	1 (0.97)	0 (0.00)	3 (2.70)	0 (0.00)	
Gender, *n* (%)					0.629
Male	32 (31.07)	26 (23.42)	28 (25.23)	115 (26.56)	
Female	71 (68.93)	85 (76.58)	83 (74.77)	318 (73.44)	
Education level, *n* (%)					0.041
High	51 (49.51)	43 (38.74)	68 (61.26)	213 (49.19)	
Medium	51 (49.51)	68 (61.26)	43 (38.74)	217 (50.12)	
Low	1 (0.98)	0 (0)	0 (0)	3 (0.69)	
Smoking, *n* (%)					0.035
Non-smoker	103 (100)	104 (93.69)	109 (98.20)	410 (94.69)	
Smoker	0 (0)	7 (6.31)	2 (1.80)	23 (5.31)	
Physical activity level, *n* (%)					<0.001
Adequate	21 (20.40)	17 (15.30)	25 (22.50)	176 (40.60)	
Low	82 (79.60)	94 (84.70)	86 (77.50)	257 (59.40)	
Body mass index (kg/m^2^), median (IQR)	30.90 (27.70–33.30)	30.70 (28.47 −34.28)	29.87 (27.69–31.88)	32.28 (29.44–35.83)	<0.001
Percentage of body fat, mean ± SD	38.41 ± 6.51^a^	39.50 ± 7.10	38.44 ± 6.15	40.52 ± 7.04^b^	0.004
Weight status, *n* (%)					<0.001
Overweight	40 (38.83)	43 (38.74)	48 (43.24)	109 (25.17)	
Obese	63 (61.17)	68 (61.26)	63 (56.76)	324 (74.83)	
Basal metabolic rate (MJ/d), median (IQR)	6.48 (5.90–7.43)	6.59 (6.03–7.21)	6.23 (5.91–6.99)	6.70 (6.10–7.60)	<0.001
Total energy intake (MJ/d), median (IQR)	8.91 (7.09–11.64)	8.98 (7.58–10.78)	8.50 (7.36–9.98)	9.29 (7.67–11.44)	0.083
EI/BMR, median (IQR)	1.35 (1.13 −1.75)	1.39 (1.18–1.68)	1.00 (1.17–1.57)	1.39 (1.14–1.66)	0.909
Energy misreporting status, *n* (%)					0.570
Plausible reporters	51 (49.5)	58 (52.3)	51 (45.9)	230 (53.1)	
Underreporters	52 (50.5)	53 (47.7)	60 (54.1)	203 (46.9)	

*BMR, basal metabolic rate; EI, energy intake; IQR, interquartile range; MJ, megajoule*.

a *n = 102*.

b *n = 430*.

**Table 2:** Food items containing walnuts at meal occasions.

**Food items at meals**	***n* (%)**
**Breakfast**	18 (14.06)
Nut, walnut, raw, unsalted	18 (14.06)
**Lunch**	13 (10.16)
Cake or cupcake, carrot, commercial, iced	1 (0.78)
Cake or cupcake, carrot, homemade from basic ingredients, undefined fat, uniced	2 (1.56)
Nut, walnut, raw, unsalted	9 (7.03)
Slice, brownie, chocolate, with nuts, homemade from basic ingredients, fat not further defined	1 (0.78)
**Dinner**	7 (5.47)
Cake or cupcake, carrot, homemade from basic ingredients, undefined fat, uniced	1 (0.78)
Nut, walnut, raw, unsalted	6 (4.69)
**Others**	90 (70.31)
Cake or cupcake, carrot, commercial, iced	4 (3.13)
Cake or cupcake, carrot, homemade from basic ingredients, undefined fat, iced	1 (0.78)
Cake or cupcake, carrot, homemade from basic ingredients, undefined fat, uniced	5 (3.91)
Nut, walnut, raw, unsalted	70 (54.69)
Slice, brownie, chocolate, with nuts, homemade from basic ingredients, fat not further defined	10 (7.81)

**Figure 1:**
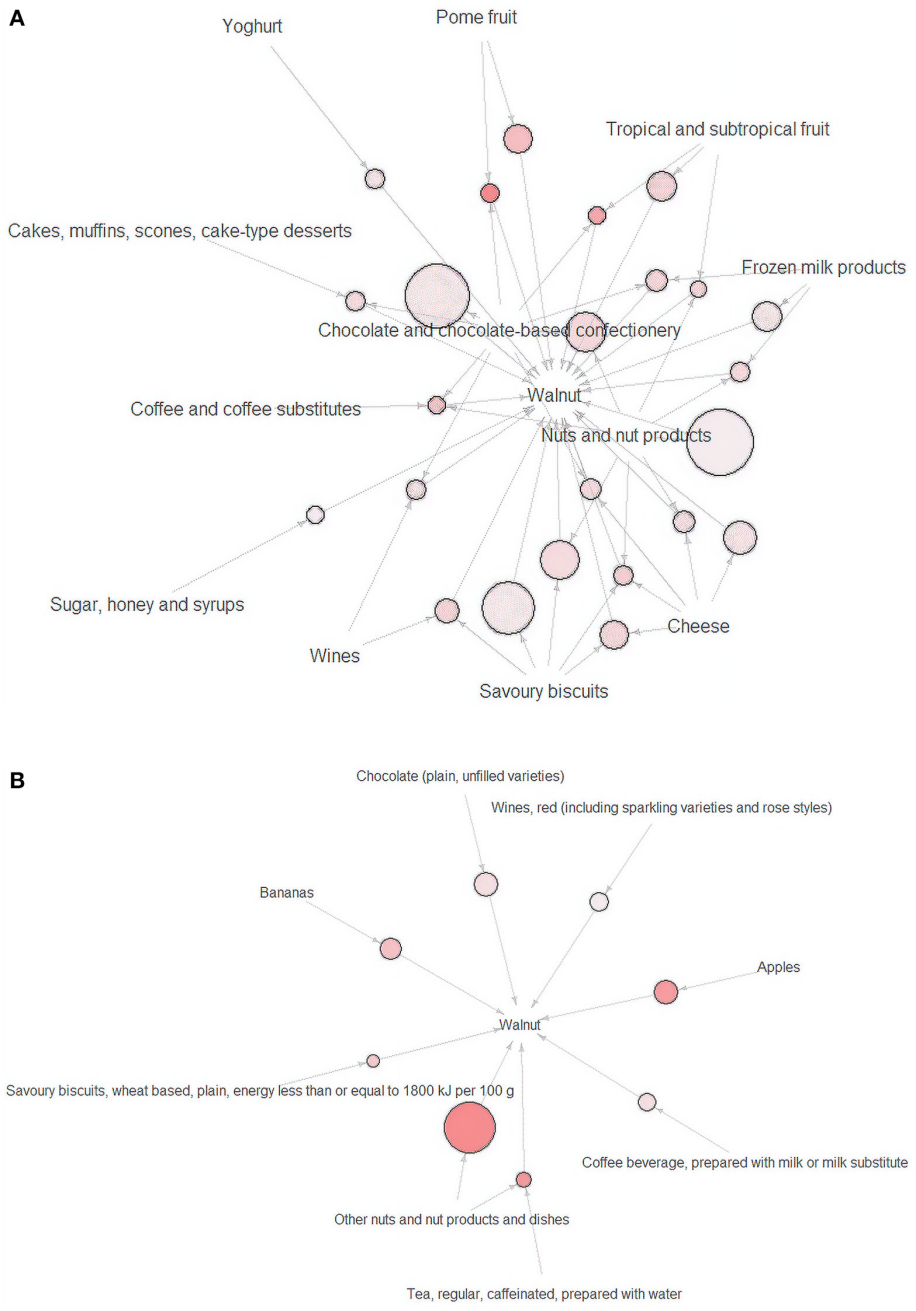
**(A)** Visualization of closely related food groups for walnuts at the submajor food group level (*n* = 24) and **(B)** visualization of closely related food groups for walnuts at the minor food groups level (*n* = 8). Arrows shows closely related food groups relationships. The size of the sphere represents the value of support. The intensity of the color indicates the value of lift.

There was no identified food choice in relation to walnut at breakfast, lunch, or dinner. A total of 24 clusters of food choices related to walnuts at other meals were identified at the submajor food group level (Figure 1A) and eight combinations at the minor food group level (Figure 1B). The details of the food choice associations with walnuts are presented in Table 3.

**Table 3:** Identified association rules between food choices and walnuts at other meals.

**Food choice 1**	**Food choice 2**	**Food choice 3**	**Walnut**	**Support**	**Confidence**	**Lift**
**SUBMAJOR FOOD GROUP LEVEL**						
Nuts and nut products			Walnut	0.6091	0.8072	1.0325
Chocolate and chocolate-based confectionery			Walnut	0.5909	0.8125	1.0392
Savory biscuits			Walnut	0.5091	0.8116	1.0381
Chocolate and chocolate-based confectionery	Nuts and nut products		Walnut	0.4273	0.8393	1.0735
Nuts and nut products	Savory biscuits		Walnut	0.4182	0.8364	1.0698
Cheese			Walnut	0.3727	0.8200	1.0488
Frozen milk products			Walnut	0.3545	0.8125	1.0392
Tropical and subtropical fruit			Walnut	0.3545	0.8478	1.0844
Cheese	Savory biscuits		Walnut	0.3455	0.8444	1.0801
Pome fruit			Walnut	0.3455	0.8837	1.1303
Savory biscuits	Wines		Walnut	0.3091	0.8500	1.0872
Cheese	Nuts and nut products		Walnut	0.3000	0.8250	1.0552
Chocolate and chocolate-based confectionery	Frozen milk products		Walnut	0.3000	0.8462	1.0823
Cheese	Chocolate and chocolate-based confectionery		Walnut	0.2909	0.8421	1.0771
Cakes, muffins, scones, cake-type desserts	Chocolate and chocolate-based confectionery		Walnut	0.2818	0.8378	1.0717
Cheese	Nuts and nut products	Savory biscuits	Walnut	0.2818	0.8611	1.1014
Chocolate and chocolate-based confectionery	Wines		Walnut	0.2818	0.8158	1.0435
Frozen milk products	Nuts and nut products		Walnut	0.2818	0.8378	1.0717
Yogurt			Walnut	0.2818	0.8158	1.0435
Chocolate and chocolate-based confectionery	Pome fruit		Walnut	0.2727	0.9375	1.1991
Chocolate and chocolate-based confectionery	Tropical and subtropical fruit		Walnut	0.2636	0.9063	1.1592
Chocolate and chocolate-based confectionery	Coffee and coffee substitutes	Nuts and nut products	Walnut	0.2636	0.8788	1.1240
Sugar, honey and syrups			Walnut	0.2636	0.8056	1.0304
Nuts and nut products	Tropical and subtropical fruit		Walnut	0.2545	0.8485	1.0853
**MINOR FOOD GROUP LEVEL**						
Other nuts and nut products and dishes			Walnut	0.5273	0.8923	1.1413
Apples			Walnut	0.3364	0.8810	1.1268
Chocolate (plain, unfilled varieties)			Walnut	0.3364	0.8222	1.0517
Bananas			Walnut	0.3182	0.8537	1.0919
Wines, red (including sparkling varieties and rose styles)			Walnut	0.3000	0.8049	1.0295
Coffee beverage, prepared with milk or milk substitute			Walnut	0.2909	0.8205	1.0495
Other nuts and nut products and dishes	Tea, regular, caffeinated, prepared with water		Walnut	0.2727	0.8824	1.1286
Savory biscuits, wheat based, plain, energy ≤1,800 kJ per 100 g			Walnut	0.2545	0.8485	1.0853

At the submajor food group level, the highest proportion of reported food choices closely related to walnuts was “nuts and nut products” (60.1%). A total of 59.1% of the participants reported having the combination of “chocolate and chocolate-based confectionary” and “walnut.” At the minor food group level, if either “other nuts and nut products and dishes,” “apples,” “chocolate (plain, unfilled varieties),” “bananas,” “wines, red,” “coffee beverage,” or “savory biscuits ≤1,800 kJ/100 g” were reported, “walnut” was also reported. If the participants reported consuming “other nuts and nuts products and dishes” and “black tea,” “walnut” was also reported (*n* = 30).

## Discussion

To our knowledge, this study is the first to report food choices in relation to walnuts in overweight and obese adult participants. By applying a novel nut food composition database, the present study has mapped the precise combinations of consumed foods associated with walnuts at mealtimes using a data mining tool. This study offers insights into the role of walnuts, which make up food choices at meals and may support nutritional guidance and dietary behavior change strategies for the intake of nuts.

Nuts are one of the key components of healthy dietary patterns (e.g., Mediterranean diet) and dietary guidelines (49, 50). They are a good source of unsaturated fatty acids and are high in fiber, vitamins, minerals, plant sterols, and polyphenol composition (25). Habitual walnut consumption has been associated with a reduced risk of cardiometabolic disease (30, 51, 52). Walnuts are high in polyunsaturated fatty acids (PUFAs) and are especially rich in α-linolenic acid and linoleic acids (25). α-Linolenic acid is an essential precursor of long-chain omega-3 PUFAs (53). The previous studies have suggested that increased level of α-linolenic acid is associated with improvements in insulin sensitivity and anti-inflammatory and antiatherogenic effects (54, 55). The unique fatty acids composition of walnut appears to play a pivotal role in the beneficial effects of cardiometabolic diseases prevention.

Adding walnut into a diet tends to improve overall dietary quality. In Australia, on average, ~35% of total daily energy consumed was from “discretionary foods” (56), which are recognized to be high in saturated fats, added sugars, salt, and/or alcohol. Low compliance with recommendations on the consumption of fruit and vegetables has remained fairly consistent over time (57). A clinical trial that provided participants with walnuts reported improvements in overall dietary quality (58). Previous research has also suggested that the provision of walnuts in the context of healthy eating advice resulted in significant increases in the quality of the overall diet patterns (59), with increased fruit consumption and decreased discretionary food consumption reported (10). Nut consumers may also have higher intakes of vegetables (60, 61). Such patterns of nut consumption, across a range of clinical trials, have demonstrated a positive association with surrogate endpoints, such as lower blood pressure and concentrations of low-density lipoprotein and triglyceride (62, 63).

However, there are many barriers to adding nuts into a diet. The current food supply is complex with a large number of foods available to the general public. Given the relative cost of consuming walnuts, food prices are the leading factor driving food decisions (64, 65), particularly for low socioeconomic status groups (66). Food literacy and skills are required to incorporate recommendations into everyday food choices practicalities (67–69). Therefore, the findings of the present study may provide examples for food selections associated with walnuts to use used in developing easier and more practical dietary strategies to improve nuts consumption.

The present exploratory cohort also suggests that walnuts were more likely to be consumed at small meals, such as midmeals or snacks. No food associations were reported at main meals, such as breakfast, lunch, and dinner. This creates an opportunity for consumer education related to the incorporation of walnuts into recipes at times when they may not be traditionally eating within the diet. Snack food choices play a vital role in the obesity epidemic along with the effects of energy imbalance (70). In Australia, there is a shift in meal patterns away from three-meals-per-day model and toward a “grazing pattern” of eating (71). This shift in meal patterns has been suggested to promote obesity (71). Particular circumstances that are also common in today's lifestyles (e.g., being rushed, having too little sleep, and experiencing psychosocial stress) can make consumers even more challenged to make healthy snack food choices (72–74). Thus, offering information on specific food choices at snacks times is required for the everyday practicalities associated with navigating the food system. The present results suggest that there were trends in food choices for walnuts at small meals in overweight and obese people. Thus, these findings may help to provide more practical dietary strategies to substitute “unhealthy” food choices with walnuts to improve overall dietary intake.

There are several strengths and limitations to the present study. The food choices associated with walnuts were generated by applying a data mining method and a nested hierarchical food grouping system that was not used in the previous study. In order to explore the food combinations associated with walnuts, detailed food choice data at meals reflecting the individual usual food consumption patterns were required. The dietary intake data used in this study was derived from a diet history interview. It uses an open-ended interview approach and probes to encourage participants to describe their usual food consumption from the first meal of the day to the end of the day (75). Although such information did focus on food choices at self-defined meals, the richness of food intake data allowed us to capture food choices association with walnuts at meal occasions (76), which may not be captured when using other dietary assessment, such as a food frequency questionnaire. While this study is considered to be one of the first food pattern applications of the *a priori* algorithm using a clinical trial dataset, there are some limitations to be acknowledged. For the purpose of the pooled data, it was assumed that all dietary data were collected in the same manner. While the dietitians involved in each of the clinical trials were all trained, it cannot be guaranteed that the procedures followed across an 11-years period did remain identical. Further, the matching of the food items in the pooled analysis was conducted by one researcher. While this was guided by a pre-constructed matching file, there may have been variations in the data over time that required subjective judgments to be made. Finally, the threshold of 25% of participants having each combination means that less common food associations may not have been captured. This specification was set for this specific analysis and should be determined based on the data characteristics and planned outcomes of the study. Nevertheless, although the health effect of walnut consumption has been frequently assessed, studies that assessed how walnuts are consumed in a diet are considerably less frequent. The pooled data analysis of clinical trials appears to offer new opportunities to complement the current evidence for the health effects of nuts by providing translational evidence toward improved the nut consumption.

In conclusion, this study offered the example of food choices associated with walnuts made by overweight and obese volunteers. It provided insights into practical strategies for improving nut intakes at the individual level. To date, less is known about the ways to incorporate walnuts into a diet during a clinical study. The present study applied a descriptive data mining tool (the *a priori* algorithm of association rules) and nested hierarchical food grouping system to examine the food choices that were associated with walnuts at meal occasions; providing data at a much deeper level. Food choices associated with walnuts at meal occasions may assist in the development of easier and more practical strategies for making food choices to improve nut consumption.

## Data Availability Statement

The data analyzed in this study is subject to the following licenses/restrictions: data can be requested from the corresponding author. Requests to access these datasets should be directed to YP, yasmine@uow.edu.au.

## Ethics Statement

The studies involving human participants were reviewed and approved by University of Wollongong Human Research Ethics Committee. The participants provided their written informed consent to participant in each trial. Consent included further use of their data for research purposes.

## Author Contributions

YP, EN, and LT: study design. VG, EN, and YP: data collation. VG: data analysis. YP, VG, and EN: data interpretation. YP, EN, and LT: funding. VG and YP: manuscript preparation. All authors contributed to the article and approved the submitted version.

## Conflict of Interest

The authors declare that previous funding has been secured from California Walnut Commission (LT), Nuts for Life (EN, LT, and YP) and the International Nut and Dried Fruit Council (EN and LT). LT and YP have previously received grant funding from NHMRC, ARC and various food industry research and development entities including Horticulture Australia Ltd. LT has previously served on the Scientific Advisory Committee of the California Walnut Commission, and currently serves in the Science Advisory Committee of the McCormick Science Institute. The remaining author declares that the research was conducted in the absence of any commercial or financial relationships that could be construed as a potential conflict of interest.
