# Supplementary material for: Identifying Usual Food Choice Combinations With Walnuts: Analysis of a 2005–2015 Clinical Trial Cohort of Overweight and Obese Adults
Source: Front Nutr. 2020 Sep 23;7:149. doi: 10.3389/fnut.2020.00149 (PMC7540216; doi:10.3389/fnut.2020.00149)
Supplement: Supplementary file 1 [file Table_1.pdf]

Supplementary Table 1: Descriptions of clinical trial studies

**Study name and number**	HELP<br>Study 1	SMART<br>Study 2	HEAL<br>Study 3	HealthTrack<br>Study 4
Registration number	ACTRN12608000453381	ACTRN12608000425392	ACTRN12610000784011	ACTRN12614000581662
Study aim	To examine the effect of energy restriction alone and with dietary fat modification on weight loss, adiposity and on risk factors for obesity related disease	To assess the effects of advice to consume two fish meals per week in a weight loss diet	To assess the effect of higher vegetable consumption on weight loss	To determine the effectiveness of a novel interdisciplinary treatment compared with usual care on weight loss
Year - baseline data collected	2005	2009	2010	2014-2015
**Inclusion criteria**				
Body Mass Index (kg/m^2)	>25	25-37	25-35	25-40
Age (years)	>18	18-60	18–65	25–54
Gender	Male and female	Male and female	Male and female	Male and female
Others	-	Waist circumference: > 94 cm (men) or > 80 cm (women),	-	Permanent residents of the Illawarra region, Australia
**Exclusion criteria**				
	• Major illnesses (e.g., cancer and diabetes)	• Major illnesses (e.g., cancer and liver diseases)	• Major illnesses (e.g., cancer and liver diseases)	• Having severe medical conditions impairing the ability to participate

	• Taking regular medication (except contraceptives)<br>• Smoking<br>• Food allergies<br>• Habits inhibiting the study<br>• Illiteracy and/or inadequate conversational English	• Diabetes mellitus<br>• Low-density lipoprotein ≥ 6mmol/L<br>• Food allergies<br>• Habits inhibiting compliance<br>• Low literacy<br>• Inadequate conversational English<br>• Those who are already taking fish oil supplements<br>• Pregnant/lactating<br>• Not weight stable (within 3 kg) in the past 6 months or on a weight-loss diet	• Diabetes mellitus<br>• Thyroid abnormalities<br>• Heavy alcohol consumption<br>• Recent acute or chronic disease<br>• Changing medications affect body weight<br>• Weight loss >5 kg in last 3 months<br>• Fluctuating exercise patterns<br>• Strenuous exercise >1 h per day<br>• Pregnancy or lactation<br>• Dietary limitations<br>• Dislike of vegetables	in the study or thought to limit survival to 1 year<br>• Being unable to communicate in English<br>• Having reported illegal drug use<br>• Regular alcohol intake associated with alcoholism (>50 g/day)<br>• Other major impediments to participation
